# Announcement: Anaheim Acs Symposium on Electron Transfer: Complementarity of Photochemistry and Radiation Chemistry in the Study of Electron Transfer

**DOI:** 10.1155/MBD.1995.187

**Published:** 1995


					ANAHEIM ACS SYMPOSIUM ON ELECTRON
TRANSFER: COMPLEMENTARITY OF
PHOTOCHEMISTRY AND RADIATION CHEMISTRY
IN THE STUDY OF ELECTRON TRANSFER
Schedule
Tutorial: Radiation Chemistry Principles and Applications
Sunday, April 2nd, 1995, 8:55 am 12:00 noon. J. F. Wishart, Presiding
8:55 James Wishart, Introductory Remarks
9:00 Helen Richter, University of Akron, Tutorial: Radiation Chemistry Principles and Applications
9:50 James Wishart, Brookhaven National Laboratory Accelerators and Other Sources for the
Study of Radiation Chemistry
10:40 Alexander Trifunac, Argonne National Laboratory Detection of Transient Species in Radiation
Chemistry
11:30 Morton Z. Hoffman, Boston University Chemistry of Radiation-Generated Intermediates
Session 1" Electron Transfer in Metalloproteins
Sunday, April 2nd, 1:25 5:30 pm., S. S. Isied, Presiding
1:25 D. Nocera and J. F. Wishart, Introductory Remarks
1:30 Harry Gray, California Institute of Technology Electron Tunneling in Proteins: Coupling
Through a 13-Strand
2:10 Israel Pecht, Weizmann Institute of Science, Israel Pathways and Control of Long-Range
Electron Transfer Within Proteins
2:50 Brian Hoffman, Northwestern University Long-Range Electron Transfer Within Protein-Protein
Complexes
3:30 James Wishart, Brookhaven National Laboratory Driving Force Dependence and
Thermodynamics of Intramolecular Electron Transfer in Ammineruthenium-Modified, Native and
Metal-Substituted Cytochromes c
4:10 Ann English, Concordia University, Canada Study of Oxyferryl Heme Reactivity using both
Radiation and Photochemical Techniques
4:50 Bill Durham, University of Arkansas Free Energy Dependence of Electron Transfer in
Cytochrome c Labeled with Ruthenium(ll) Polypyridine Complexes
Session 2: Electron Transfer in Bridged Donor-Acceptor Systems
Monday, April 3rd, 1:30 5:30 pm., J. F. Wishart, Presiding
1:30 Stephan Isied, Rutgers, the State University of New Jersey Electron Transfer Across Peptide
and Protein Matrices: Using the Full Time Domain of the Pulse Radiolysis Techniques
2:10 George McLendon, University of Rochester Photochemistry and Radiation Chemistry Studies
of Intra Protein and Intra Peptide Electron Transfer
2:50 Krzysztof Bobrowski, Institute of Nuclear Chemistry and Technology, Warsaw, Poland Long
Range Electron Transfer Between Proline-Bridged Aromatic Amino Acids
3:30 John Miller, Argonne National Laboratory Pulse Radiolysis Measurements of Intramolecular
Electron Transfer
4:10 Thomas Moore, Arizona State University Photoinduced Electron Transfer and Proton Transfer
in a Molecular Triad
4:50 Michael Wasielewski, Argonne National Laboratory Bridged Donor-Acceptor Molecules that
Mimic the Radical Pair and Triplet States of Photosynthetic Reaction Centers
187
Session 3: Small-Molecule Electron Transfer and Activation
Wednesday, April 5th, 8:40 am 12 noon., R. van Eldik, Presiding
8:40 Diane Cabelli, Brookhaven National Laboratory Kinetic Studies of Superoxide with Manganese
Complexes/Manganese Superoxide Dismutase
9:20 Klaus-Dieter Asmus, Hahn-Meitner-lnstitute, Berlin, Germany and the Notre Dame Radiation
Laboratory Intermediates in the Oxidation of CuI(TTCN)2 in Aqueous Solution
10:00 Carol Creutz, Brookhaven National Laboratory Mechanisms of Photochemical Reactions
Elucidated by Pulse Radiolysis Experiments
10:40 James Espenson, Ames Laboratory, Iowa State University Metal Radicals and Other Free
Radicals: Electron Transfer Reactions
11:20 Noel Hush, University of Sydney, Australia The Primary Process in Photooxidation of Fe2+ in
Water
Session 4: Electron Transfer in Materials, Unusual Conditions and Ultrashort Timescales
Wednesday, April 5th, 1:30 5:35 pm., D. Nocera, Presiding
1:30 Morton Z. Hoffman, Boston University Photoinduced Reduction of Tris(2,2'-
bipyrazine)ruthenium(2+) Ion in Aqueous Solution
2:10 Jacqueline Belloni, Universit de Paris-Sud, France Kinetics of Electron Transfer Catalyzed by
Metal Clusters
2:50 John Warman, Technical University of Delft, Netherlands Charge Migration and
Recombination in Pulse-Irradiated Columnar Aggregates of Porphyrins and Phthalocyanines
3:30 Nicholas Turro, Columbia University Electron Transfer on Starburst Dendrimers and the Use of
DNA as a Molecular Wire
4:10 Rudi van Eldik, University of Erlangen, Germany Electron Transfer Reactions Under High
Pressure: Application of Spectroscopic and Electrochemical Techniques
4:50 Yann Gauduel, Laboratoire d'Optique Applique, Ecole Polytechnique ENSTA, Palaiseau,
France Ultrashort-Lived Pre-Chemical Steps in Aqueous Ionic Solutions
5:30 D. Nocera and J. F. Wishart, Closing Remarks
Session 5" Contributed Talks
Thursday, April 6th, 9:00 am 11:40., D. Nocera, Presiding
9:00 Savitri Chandrasekhar, University of Saskatchewan Pulse Radiolytic Studies of the Redox
Reactions of Pt(ll) Macrocyclic Complexes
9:20 Etsuko Fujita, Brookhaven National Laboratory Toward Photoreduction of CO2 with Ni(bpy)n2+
Complexes
9:40 Steven W. Keller, Penn State University Photochemically Induced Charge Separation in
Electrostatically Constructed Organic-Inorganic Multilayer Composites
10:00 James A. Roberts, Michigan State University Photoinduced Electron Transfer Between an
Acceptor Donor Pair Juxtaposed by a Salt Bridge
10:20 Piotr Piotrowiak, University of New Orleans Electrolyte Effects in Intramolecular Electron
Transfer
10:40 H. Holden Thorp, University of North Carolina Electron Transfer Reactions Between DNA and
Metal Complexes: Model Studies for Ionizing Radiation Damage
11:00 David Wiedenfeld, California Institute of Technology Isotope Effects and Temperature
Dependence of Intramolecular Electron-Transfer Reactions
11:20 Jeffrey Regan, University of California at San Diego Simple Theoretical Model to Predict the
Influence of the Protein Environment on Tunneling Matrix Elements
188

				

